# Comparison of ultrasound-guided closed reduction and percutaneous pinning fixation for unstable humeral lateral condylar fractures

**DOI:** 10.3389/fsurg.2024.1392910

**Published:** 2024-05-10

**Authors:** Jianbing Xu, Chaoyu Liu, Guoqiang Jia, Xiuming Huang

**Affiliations:** ^1^ Department of Orthopaedics, Ganzhou Maternal and Children’s Health Care Hospital, Ganzhou, China; ^2^ Department of Orthopaedics, Fuyang People’s Hospital of Anhui Medical University, Fuyang, China; ^3^ Department of Orthopaedics, Children’s Hospital of Fudan University Anhui Hospital, Hefei, China

**Keywords:** ultrasound-guided, song classification, closed reduction, lateral condyle, children, humeral

## Abstract

**Objective:**

Ultrasound-guided techniques have become popular in severe humeral lateral condylar fractures (HLCFs). This study compared the results of ultrasound-guided closed reduction and percutaneous pinning (UG-CRPP) for Song types 4 and 5 and dislocation type of HLCFs.

**Methods:**

This retrospective study was conducted in patients with HLCFs treated between January 2021 and October 2022 at three hospitals. The patients were divided into three groups according to Song's classification and elbow dislocation. The surgical time, reduction failure rate, and outcomes of the three groups were compared.

**Results:**

The mean surgical time of the 94 patients across the three groups (Song 4 group, 42 cases; Song 5 group, 38 cases; and dislocation group, 14 cases) was the longest for Song 4 (66.14 ± 23.05 min), followed by the dislocation group (59.71 ± 21.07 min) and Song 5 (52.16 ± 14.94 min) (for all, *P* = 0.009). The failure rate decreased in the following order: dislocation group (5/14), Song 4 group (7/42), and Song 5 group (2/38). The failure rate of closed reduction in Song 4 was 3.2-fold higher than that in Song 5, and for the dislocation group, it was 7.6-fold higher than that in Song 5. Significant differences were observed between the Song 4, Song 5, and dislocation groups in terms of shaft-condylar angle and supination (*P* = 0.015, *P* = 0.043). No significant differences (*P* > 0.05) were observed in the carry angle, flexion, extension, or pronation of the three groups. Two cases of delayed healing, four cases of superficial infection, one case of trochlear necrosis, and 39 cases of lateral spur in the Song 4 group were observed. In the Song 5 group, five had a superficial infection, one had re-displacement, and 26 had a lateral spur. In the dislocation group, there were two cases of superficial infection and 10 of lateral spurs.

**Conclusions:**

Song 4 HLCFs require longer surgical time and present more postoperative complications than Song 5 and dislocation-type HLCFs and can easily lead to lateral spurs. The failure rates of closed reduction in Song 4 and the dislocation type were higher than those in Song 5. Thus, UG-CRPP can be used to treat patients with unstable HLCFs.

## Introduction

Humeral lateral condylar fractures (HLCFs) are the second most common type of elbow fracture in children (1). HLCFs often involve the distal humeral cartilage, and radiographs cannot reveal the alignment of the cartilage hinge, either during diagnosis or treatment. In 2008, Song proposed a novel classification method based on fracture line characteristics and a treatment algorithm (2). Types 1–2 are treated conservatively with plaster fixation, while types 3–5 first undergo closed reduction; if closed reduction fails, open reduction is recommended (2, 3). Currently, ultrasound-guided closed reduction and percutaneous pinning (UG-CRPP) fixation option is becoming popular and widely performed to treat HLCFs because it provides non-radiative, multi-directional dynamic monitoring of the CRPP process and bilateral control scanning (4–8). Compared with open reduction, first of all, it is minimally invasive and aesthetically pleasing. Secondly, it does not damage the common tendon of the lateral extensor muscle, preventing a decrease in muscle strength. Thirdly, it protects the capitellum and reduces its impact on blood circulation. However, no reports have described the outcomes of HLCFs treated with UG-CRPP, including dislocation-type HLCFs. Therefore, we hypothesized that patients with different Song HLCF subtypes following UG-CRPP had different outcomes.

## Methods

### Clinical data

We retrospectively collected data from patients with Song types 4 and 5 and dislocation type of HLCFs admitted to three different hospitals between January 2021 and October 2022.

### Inclusion and exclusion criteria

The inclusion criteria were as follows: (1) Song classification of unstable type (Song 4, Song 5, and dislocation type), (2) age <14 years, and (3) follow-up period >6 months. The following cases were excluded: (1) HLCFs combined with other types of fractures in the same limb, (2) open and pathological fractures, (3) missing ultrasound imaging, and (4) incomplete clinical data.

This study was approved by the ethics committees of the three hospitals. All parents or patients' guardians signed informed consent forms.

### Surgical technique

**Figure 1:**
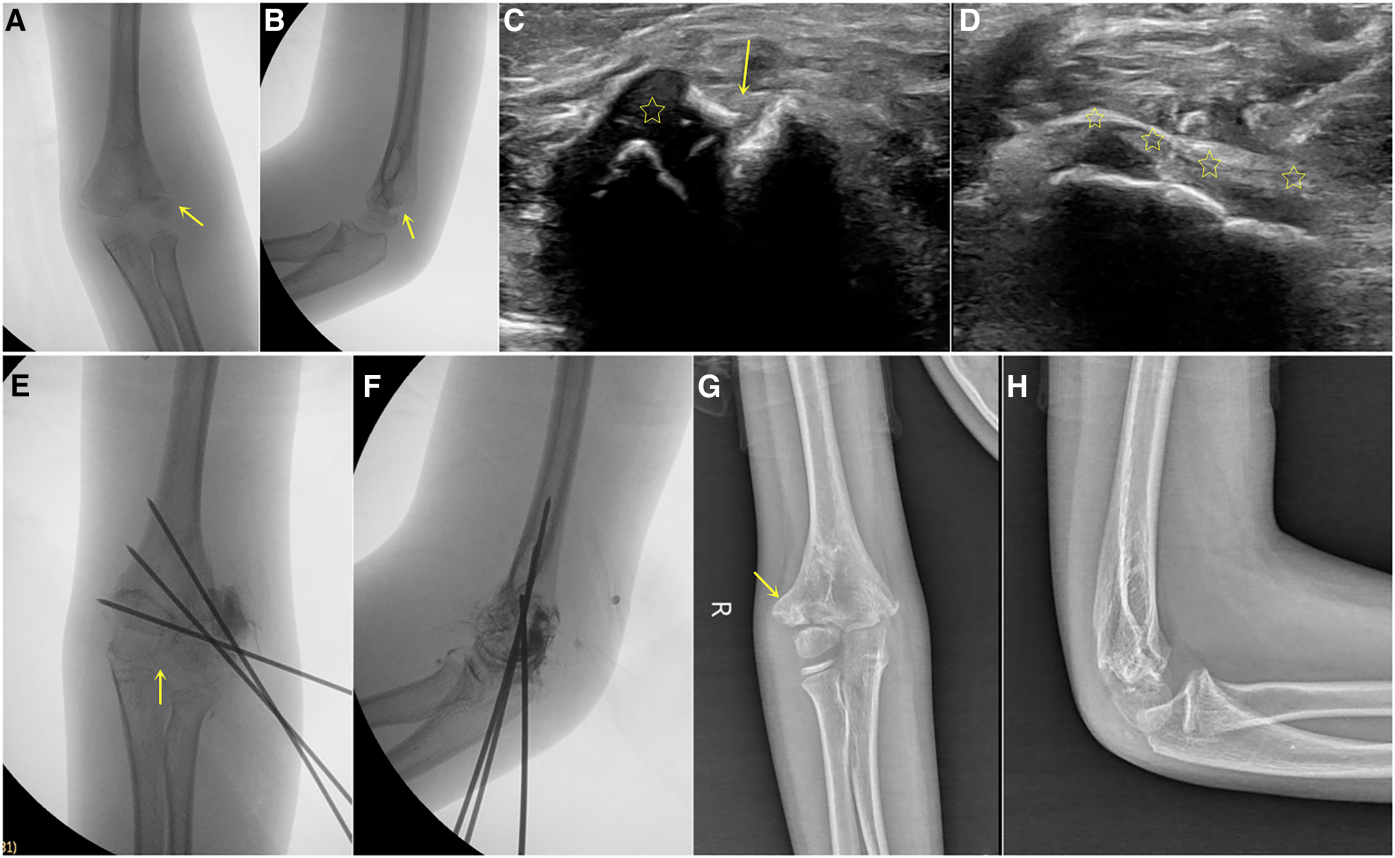
Boy, 8 years old, with right humeral lateral condyle fracture, song type 4. (**A,B**), preoperative x-ray showed lateral displacement of the fracture, with obvious displacement but no rotated (arrow). (**C**) Preoperative ultrasound showed displacement of fragment (arrow indicates fracture line, asterisk indicates fracture fragment). (**D**) After reduction, continuous cartilage hinges were observed (arrow). (**E,F**) Postoperative imaging showed continuous cartilage hinge (arrow) and divergent Kirschner wires fixation. (**G,H**) After 20 months of postoperative follow-up, obvious lateral spur (arrow) was observed, and the elbow function was good.

**Figure 2:**
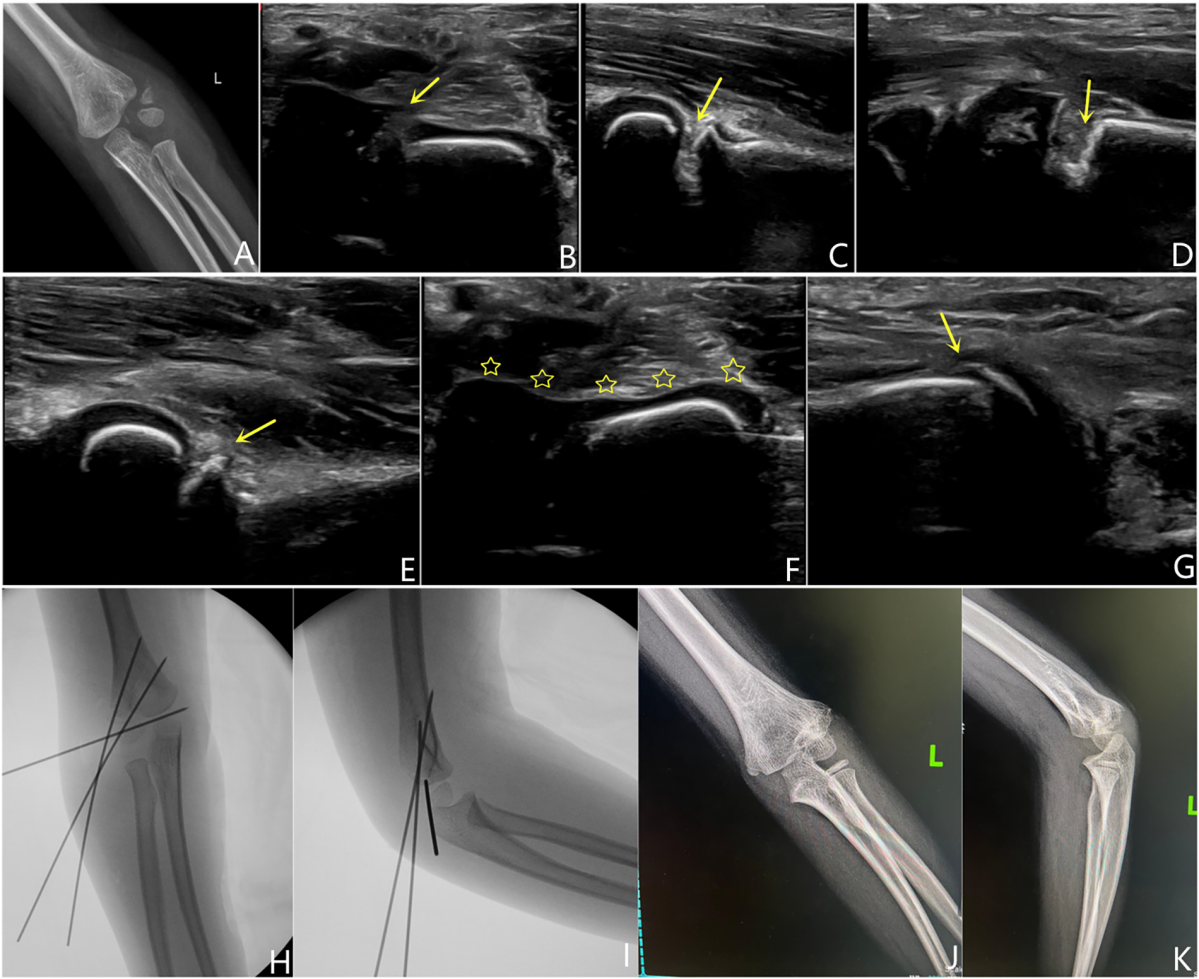
Boy, 5 years old, with left humeral lateral condyle fracture, song type 5. (**A**) Preoperative x-ray showed a rotated fragment (arrow). (**B–D**) Before reduction, severe displacement of the fracture can be seen on ultrasound in the coronal, sagittal, and metaphyseal directions (arrow). (**E–G**) After reduction and fixation, fracture reduction can be seen in the sagittal, coronal, and metaphyseal directions on ultrasound, with continuous cartilage hinge. (**H,I**) Postoperative imaging showed continuous cartilage hinge and divergent Kirschner wires fixation after reduction. (**J,K**) After 21 months of surgery, the follow-up pictures showed that the fracture were normal-like shape with minor lateral spur.

**Figure 3:**
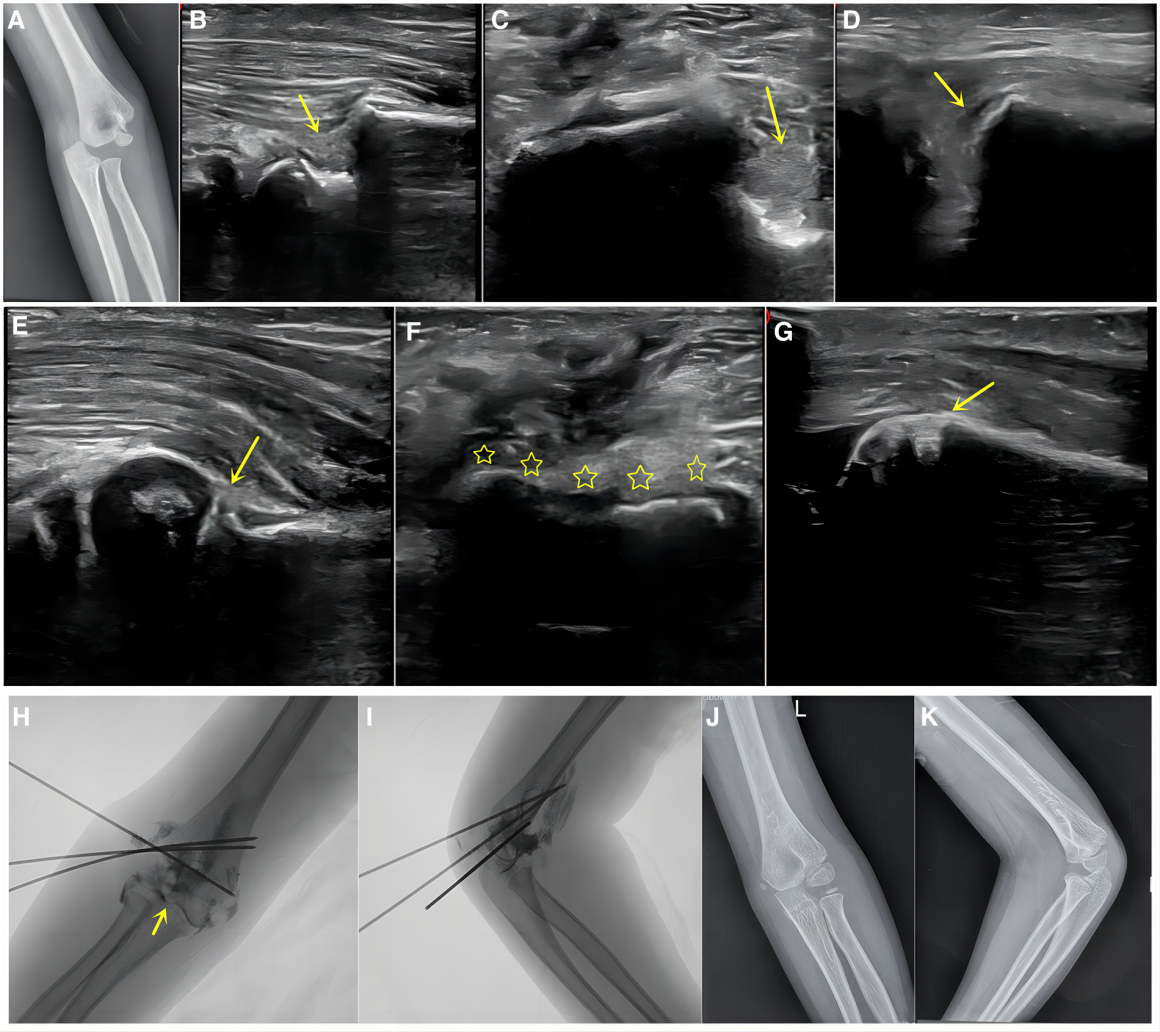
Boy, 6 years old, left humeral lateral condyle fracture, dislocation type. (**A**) Preoperative x-ray revealed dislocation of the elbow joint with a rotated fragment. (**B–D**) Before reduction, severe displacement can be seen on ultrasound in the sagittal, coronal, and posterior lateral metaphyseal directions (arrow). (**E–G**) After reduction and fixation, good reduction can be seen in the sagittal, coronal, and posterior lateral metaphyseal directions on ultrasound, with continuous cartilage hinge and normal condylar-shaft angle. (**H,I**) Postoperative imaging showed continuous cartilage hinge after reduction (arrow), divergent Kirschner wires fixation. (**J,K**) After 21 months of postoperative follow-up, it was found that the fracture was normal, without lateral spur and the function was excellent.

The same surgical techniques were used in all three hospitals by three attending doctors (JBX, CYL, and GQJ). The procedure was performed in the supine position, with the affected arm placed on a C-arm platform. For Song 4, the affected arm was gently tracked in a straight position, and varus the elbow to create a space for fragment reduction. The reduction quality was assessed using coronal transverse, coronal anterolateral longitudinal, sagittal lateral longitudinal, and sagittal posterolateral longitudinal scans (Figure 1). For Song 5, the varus was applied first to the affected arm, and the thumb was then placed between the gap of the two fragments; second, the valgus and flexion were applied to the elbow; and third, the elbow alignment was checked by ultrasound (Figure 2). If the reduction failed, a 2.0-mm K-wire was interposed into the fragment gap by prying to de-rotate and reduce the fragment (9). With dislocation-type HLCFs, the elbow was first reduced, followed by the procedure used for Song type 5 (Figure 3). After successful reduction, three K-wires were fixed divergently from the cartilage to the metaphysis, for children over 7 years old, we choose a 2.0 mm Kirschner wire, and for children under 7 years old, we choose a 1.5 mm Kirschner wire, followed by a long-arm cast in a neutral position.

### Evaluation of perioperative and follow-up results

The hardware was removed 6 weeks after surgery. The surgical time was recorded, and radiography was performed on the affected side to assess the Baumann angle, condylar shaft angle, carry angle, and lateral spur at the latest follow-up. The lateral spur was evaluated using the intercondylar width ratio, and the function of the elbow joint was evaluated using the Mayo standard (10, 11). Infection, nonunion, cubitus varus, and vagus deformities were analyzed.

### Statistical analysis

Statistical analyses were performed using IBM SPSS software (v.23.0; IBM Corp., Armonk, NY, USA). Continuous variables were represented by the mean ± standard deviation. A one-way analysis of variance was used for comparisons between the three groups. Statistical significance was set at *P* < 0.05.

## Results

### Patient characteristics and clinical outcomes

A total of 157 cases were enrolled in this study. After applying the exclusion criteria, 94 patients were included [63 male and 31 female; mean age, 4.81 years (1–11 years)]. The mean time from injury to surgery was 1.83 days (1–15 days). Forty-four cases were left-sided, and 50 cases were right-sided. Forty-two patients were in the Song 4 group, 38 in the Song 5 group, and 14 in the dislocation group.

The sequence of mean surgical time was in the following order: Song 4 (66.14 ± 23.05 min) >dislocation group (59.71 ± 21.07 min) >Song 5 (52.16 ± 14.94 min), with significant differences among groups (*F* = 4.955, *P* = 0.009). The failure rate of UG-CRPP was, in order, Song 5 (2/38) <Song 4 (7/42) <dislocation group (5/14). The odds ratio of the failure rate of Song 4 UG-CRPP was 3.2-fold that of Song 5, and that of the dislocation group was 7.6-fold that of Song 5 (7/42). Perioperative and follow-up results are compared in Table 1. Typical cases are shown in Figures 1–3. No statistically significant differences were observed for the Mayo score of the elbow among the three groups.

**Table 1:** Clinical and radiographic outcomes.

Measurements and parameters	Song 4 ($\bar{x}$, range)	Song 5 ($\bar{x}$, range)	Dislocation ($\bar{x}$, range)	*F*	*P*
Sample size (*n*)	42	38	14	/	/
Mean age (years)	4.88 (2–9)	4.53 (1–11)	5.36 (2–9)	0.91	0.41
Mean time (min)	66.14 (26–137)	52.16 (26–108)	59.71 (28–114)	4.955	0.009
Follow-up (months)	13.64 (6–23)	19.64 (6–22)	15.29 (11–21)	1.425	0.246
Failure rate (%)	16.67	5.26	35.7	–	–
Carry angle (°)	7.18 (1–13)	8.42 (2–17)	8.76 (4–15)	1.786	0.173
Condylar shaft (°)	42.11 (21–50)	38.83 (24–47)	36.85 (29–44)	5.499	0.006
Baumman angle (°)	71.19 (62–86)	72.42 (63−82)	73.57 (68–79)	1.192	0.308
Extension (°)	4.76 (−5–20)	5.63 (−5–15)	7.14 (5–10)	1.398	0.252
Flexion (°)	139.4 (120–155)	136.8 (120–150)	140 (130–150)	1.921	0.152
Pronation (°)	86.79 (80–90)	86.32 (80–90)	85.36 (80–90)	0.942	0.393
Supination (°)	88.45 (85–90)	86.97 (80–90)	86.86 (80–90)	1.920	0.153
Mayo score (points)	88.40 (80–95)	90.34 (80–95)	90.71 (85–100)	0.498	0.610

### Outcomes

In the two-group comparisons, there was a significant difference in the condylar shaft angle between the Song 4 and 5 groups (*P* < 0.05) and between the Song 4 and dislocation groups. Supination differed significantly between the Song 4 and 5 groups (*P* = 0.043). There were no statistically significant differences (*P* > 0.05) in the Baumann angle, carry angle, elbow joint activity, or rotation. Table 1 summarizes the results of the study.

### Complications

In the Song 4 group, two patients presented delayed healing, four developed a superficial infection, one showed trochlear necrosis, and 39 presented with a lateral spur. In the Song 5 group, one patient presented with malunion, five had a superficial infection, one presented with a re-displacement, and 26 had a lateral spur. In the dislocation group, two cases developed a superficial infection, and 10 had a lateral spur. None of the patients developed a cubitus varus or deep infection. Complications are listed in Table 2.

**Table 2:** Complications stratified by HLCFs group.

Complications	Song 4 (*n*)	Song 5 (*n*)	Dislocation type (*n*)
Delayed union	2	1	0
Necrosis	1	0	0
Infection	4	5	2
Lateral spur	39	26	10
Re-displaced	0	1	0

HLCFs, humeral lateral condylar fractures.

## Discussion

HLCFs require anatomical reduction to ensure elbow function. For unstable HLCFs, conventional open reduction results in lower patient satisfaction owing to an obvious scar on the lateral side of the elbow. Currently, the UG-CRPP technique has been increasingly applied to elbow fractures (4–8, 12). The advantages of ultrasound include the multidirectional display of the cartilage hinge position, fragment shape, rotation, and alignment; the real-time alignment of the two parts can be displayed during the reduction process, which allows the cartilage fixation process to be supervised, reducing the number of pin penetrations for accurate fixation and premature physeal damage (PPD). In the patients included in this series, fixation was only performed in the metaphyseal without crossing the physis, which further reduced the PPD. Among the 94 patients in the three groups, different types of fractures achieved different treatment outcomes, indicating that the fracture type affected the outcomes.

The order of failure rates for the three groups of UG-CRPP fractures was dislocation-type, Song 4, and Song 5 HLCFs. There are multiple possible reasons. (1) Anatomically, the Song 4 fracture fragment does not flip and manifests as a posterolateral displacement. Under these conditions, the fragment was small, was difficult to control during reduction, and could easily be displaced when the thumb was pressed heavily. (2) In terms of injury violence, dislocation-type injuries derive mostly from relatively heavier trauma, and the fracture line usually passes through the outer edge of the trochlear spine. The fragment was less stable and prone to re-dislocation, leading to the highest UG-CRPP failure rate. Thus, there is a situation of reduction-re-dislocation-reduction during surgery of the dislocation type, which makes controlling the reduction force more difficult. In Song 5 HCLFs, the violence of the injury was greater than that in Song 4, and even when the fracture fragment was flipped, there was a large space for reduction. When the flipped fragment is returned, it becomes stable and easy to control. Once the fragment is reduced, an anatomical reduction can be almost achieved with a certain degree of thumb compression. (3) In terms of treatment techniques, an extended position is generally used for reduction. For Song 5 and dislocation-type HLCFs, the fragment was generally displaced posterolaterally, whereas Song 4 was mainly displaced laterally. (4) In the soft tissue, the lateral condyle serves as the insertion point for the common extensor tendon and pronator teres muscles. When the fragment flips over, the force of the soft tissue obstruction factor is released, making it easier to reduce. In Song 4, the soft tissue was pulled outward and backward, which mainly revealed a lateral displacement and required a slight heave force to resist reduction.

A comparison of closed and open reductions for unstable HLCFs has confirmed the feasibility and effectiveness of UG-CRPP (6, 8, 13–15). Although open reduction can remove blood scabs or other incarcerated soft tissues, the overall incidence of complications between closed means is similar (13–15).

This three-center study with a medium sample size included all severely displaced HLCFs and an uncommon type of dislocation with more comprehensive fracture characteristics. It clearly reported the treatment outcomes of severely displaced HLCFs compared to previous studies (16–18). The surgical time was not increased, and the incidence of complications was comparable to that of previous reports (8, 19–21). No significant differences in complications between the three groups were observed. One patient in the Song 5 group exhibited mild postoperative re-displacement; the patient was a toddler with less stable fixation. Therefore, we advocate using three divergent K-wires for fixation to reduce the possibility of re-displacement. One patient with Song 4 had a fragmented ossification nucleus in two parts. Further follow-up is needed to observe the development of the ossification nucleus. All three groups had a high incidence of moderate lateral spurs; some patients even presented with medial spurs, but there were almost no functional limitations. In practice, we advocate fixing a K-wire in the physis and two others in the metaphysis from the posterolateral to anterior-medial to avoid penetrating the physis, as with techniques like screw fixation (22). We also recommend screw fixation after 8 years of age in type 4 patients, as compression fixation can promote bone healing and reduce lateral spur formation.

Cases of UG-CRPP failures at the three centers occurred in the first 3 months of adopting this surgical technique. As the surgeon's experience increased, the number of patients requiring open reduction rapidly decreased, indicating that after a certain learning curve, the surgical technique matured further and outcomes were further optimized.

This study had some limitations. First, this was a retrospective study with a high missed follow-up rate, which adds to the results bias. Second, this study subtyped fractures, resulting in a small sample size for each group, especially for dislocations, which reduced the effectiveness of the subtype comparison. Finally, only the contralateral function was compared, with no comparison of any radiographic parameters.

## Conclusion

Patients with Song 4-type HLCFs have a longer surgical time and more postoperative complications than those with other HLCFs and have a lateral spur. Additionally, the failure rates of the UG-CRPP in the dislocation type and Song 4 are higher than those with Song 5 fractures.

## Data Availability

The raw data supporting the conclusions of this article will be made available by the authors, without undue reservation.
